# Baseline pressure errors (BPEs) extensively influence intracranial pressure scores: results of a prospective observational study

**DOI:** 10.1186/1475-925X-13-7

**Published:** 2014-01-28

**Authors:** Per Kristian Eide, Angelika Sorteberg, Torstein R Meling, Wilhelm Sorteberg

**Affiliations:** 1 Department of Neurosurgery, Oslo University Hospital - Rikshospitalet, Oslo, Norway; 2 Faculty of Medicine, University of Oslo, Oslo, Norway

## Abstract

**Background:**

Monitoring of intracranial pressure (ICP) is a cornerstone in the surveillance of neurosurgical patients. The ICP is measured against a baseline pressure (i.e. zero - or reference pressure). We have previously reported that baseline pressure errors (BPEs), manifested as spontaneous shift or drifts in baseline pressure, cause erroneous readings of mean ICP in individual patients. The objective of this study was to monitor the frequency and severity of BPEs. To this end, we performed a prospective, observational study monitoring the ICP from two separate ICP sensors (Sensors 1 and 2) placed in close proximity in the brain. We characterized BPEs as differences in mean ICP despite near to identical ICP waveform in Sensors 1 and 2.

**Methods:**

The study enrolled patients with aneurysmal subarachnoid hemorrhage in need of continuous ICP monitoring as part of their intensive care management. The two sensors were placed close to each other in the brain parenchyma via the same burr hole. The monitoring was performed as long as needed from a clinical perspective and the ICP recordings were stored digitally for analysis. For every patient the mean ICP as well as the various ICP wave parameters of the two sensors were compared.

**Results:**

Sixteen patients were monitored median 164 hours (ranges 70 – 364 hours). Major BPEs, as defined by marked differences in mean ICP despite similar ICP waveform, were seen in 9 of them (56%). The BPEs were of magnitudes that had the potential to alter patient management.

**Conclusions:**

Baseline Pressure Errors (BPEs) occur in a significant number of patients undergoing continuous ICP monitoring and they may alter patient management. The current practice of measuring ICP against a baseline pressure does not comply with the concept of State of the Art. Monitoring of the ICP waves ought to become the new State of the Art as they are not influenced by BPEs.

## Background

Continuous intracranial pressure (ICP) monitoring is a cornerstone in the surveillance of patients suffering traumatic brain injury or intracranial hemorrhage [1-4]. ICP can be measured either using solid ICP sensors placed in the brain parenchyma, or via a fluid catheter (external ventricular drain; EVD) placed within a cerebral ventricle. To protect patients from brain damages secondary to pathologically raised ICP, the one management goal is usually to keep the static pressure parameter “mean ICP” <20-25 mmHg [3], or the dynamic pressure parameter “mean ICP wave amplitude” (MWA) <5 mmHg [1].

The mean ICP is calculated against a baseline reference pressure, also denoted the zero pressure level. If this baseline reference pressure becomes altered, the ICP value displayed to the physician will be erroneous [5]. Such baseline pressure errors (BPEs) occur during clinical ICP monitoring [5,6], and may occur with various types of ICP sensors [6].

However, despite of an erroneously measured static mean ICP, the sensor’s ability to read swift changes in ICP remains intact. Consequently, although the recorded static ICP has become erroneous, i.e. the dynamic ICP remains unaltered. A BPE thus characteristically alters the mean ICP, making it erroneous, but leaves the MWA unchanged.

If a patient is monitored with just one ICP sensor, it is difficult to identify BPEs unless the MWA is recorded simultaneously. However, the impact of BPEs on the mean ICP may be studied if the ICP is recorded simultaneously from two separate ICP sensors in close proximity within the same cerebral hemisphere. BPEs will then be revealed as differences occurring in mean ICP without a concomitant changes in various ICP waveform parameters such as the MWA and the mean wave rise time (MWRT) (dynamic pressure parameter) between the two sensors [5].

In this prospective study on 16 patients with aneurysmal subarachnoid haemorrhage (SAH), we placed two separate ICP sensors in the brain parenchyma via the same burr hole, and recorded the ICP simultaneously from the two sensors in order to determine the frequency and severity of BPEs.

## Methods

### Patients

The study enrolled patients with aneurysmal SAH in need of continuous ICP monitoring as part of their intensive care management. Inclusion in the study did not otherwise influence patient management.

The Regional Ethics Committee, REK South-East (2010/1328B) and Oslo University Hospital (2010/16315) approved this study. Inclusion was by written and oral informed consent, either by the patient herself/himself or by the closest family member.

### Study design

The study design was prospective observational. The sole objective was to determine the frequency and severity of BPEs. It was thus beyond the scope of the study to explore how BPEs may affect outcome and the clinical decision making process.

### Monitoring and analysis of ICP

The two ICP sensors, both Raumedic NeuroVent P (Raumedic AG, Münchberg, GE), were placed in the brain parenchyma via the same burr hole, either during aneurysm surgery, or during placement of an EVD. The location of the ICP sensors was verified by cerebral computer tomography (CT) scanning.

The sensors were connected to the MPR-1 monitor (Raumedic AG, Münchberg, GE), which in turn was connected to a laptop computer running Sensometrics Software (dPCom AS, Oslo, Norway). Sampling of pressure signals from the two sensors (Sensors 1 and 2) was performed with a digital sampling rate of 100 Hz. The raw data files were stored on the computer. Recordings from the two sensors continued throughout the time period the patient was clinically deemed to be in need of ICP surveillance.

**Figure 1:**
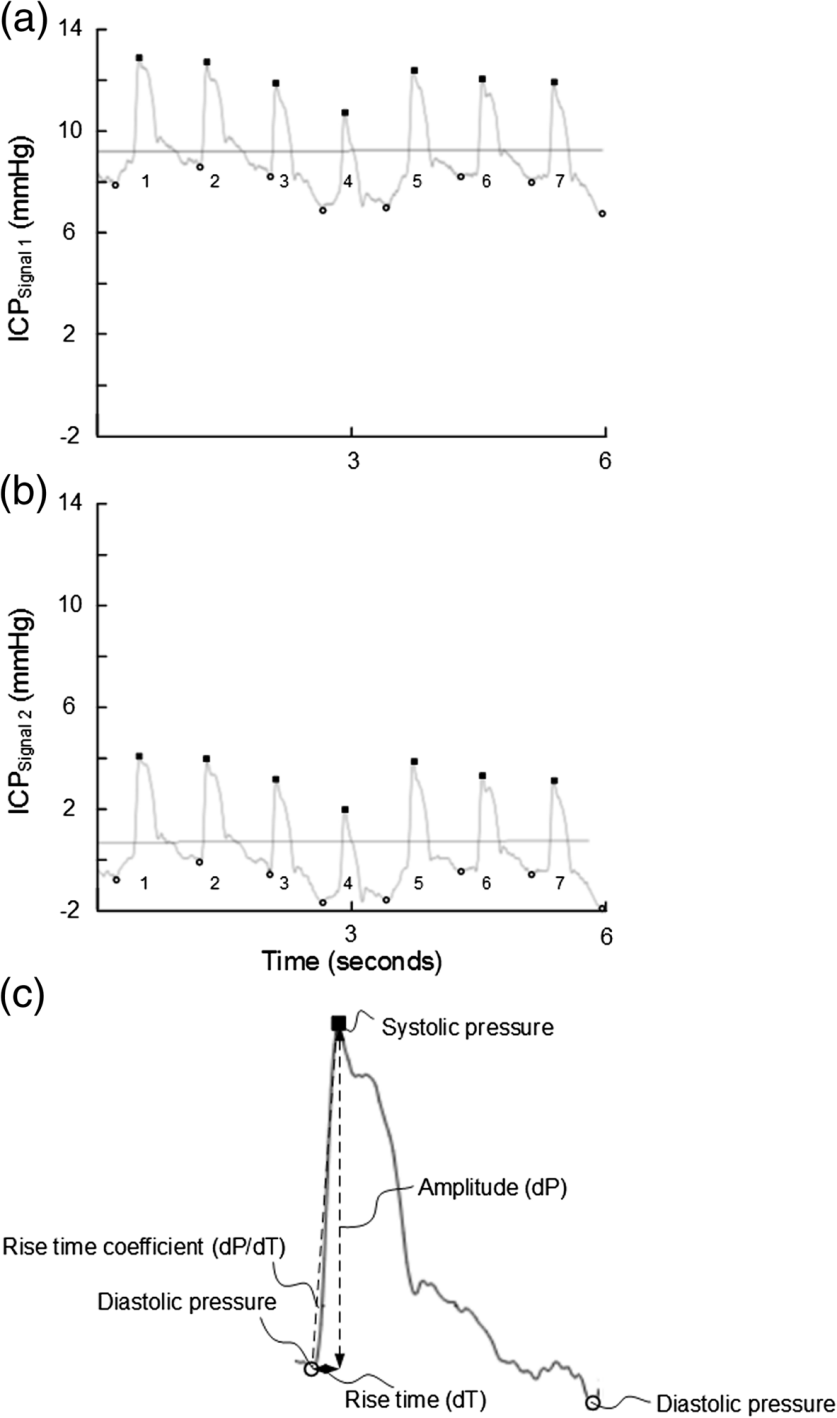
**Simultaneous 6-sec time windows of signals 1 and 2.** An example of a 6-sec time window showing the signal of **(a)** ICP Signal 1 and **(b)** ICP Signal 2. The 6-sec time window contains seven cardiac-induced ICP waves; the amplitude (dP), rise time (dT) and rise time coefficient (dP/dT) were automatically determined for each wave **(c)**. The mean ICP (horizontal lines) and mean ICP wave amplitude (MWA) were determined for every consecutive 6-sec time window. For the 6-sec time window shown in a-b, the values were as follows: **(a)** Signal 1: Mean ICP_Signal 1_ 9.0 mmHg, MWA_Signal 1_ 4.3 mmHg, MWRT_Signal 2_ 0.23 sec, and MWRTC_Signal 2_ 24.1 mmHg/sec. **(b)** Signal 2: Mean ICP_Signal 2_ 0.3 mmHg, MWA_Signal 2_ 4.3 mmHg, MWRT_Signal 2_ 0.24 sec, and MWRTC_Signal 2_ 24.2 mmHg/sec. For this particular 6-sec time window, the BPE was represented by a difference in mean ICP of 8.7 mmHg, despite close to identical waveform, represented by difference in MWA of 0.05 mmHg, difference in MWRT of 0.003 sec, and difference in MWRTC of 0.14 mmHg/sec.

The ICP signals were analyzed according to the methodology implemented in Sensometrics Software (Figure 1) [7]. In short, the heartbeat-induced single ICP waves were identified and differentiated from pressure waves of other origins (noise or various artifacts). For each heart-beat-induced single ICP wave the following wave parameters were determined: the amplitude (dP), rise time (dT), and the rise-time coefficient (dP/dT) (Figure 1). Only 6-sec time windows containing a minimum of four heartbeat-induced waves were included for further analysis. Based on the single ICP wave parameters, the following 6-sec single wave indices were determined: the MWA, the mean wave rise time (MWRT), and the mean wave rise time coefficient (MWRTC) (Figure 1).

The parameter “mean ICP” is independent of single ICP wave identification and was determined for every consecutive 6-sec time window as the sum of sample values divided by the number of samples. 6-sec time windows showing differences in mean ICP between the two sensors in excess of 50 mmHg were excluded from analysis as we hypothesized that such a large shift in mean ICP in a clinical setting would be detected and the recording discarded, and are hence of little relevance for a study as performed here.

For every consecutive 6-sec time window, differences in mean ICP were determined for Sensors 1 and 2. For accepted 6-sec time windows wherein MWA, MWRT, MWRTC were determined, differences in parameter-values between Sensors 1 and 2 were determined (Figure 1).

### Determination of BPEs

The traditional way of computing mean ICP is shown in formula (1).

(1) $$\mathrm{ICP}=P_{\mathrm{Intracranial}}-P_{\mathrm{Baseline}}$$

A requirement for correct ICP measurement is that P_Baseline_ equals 0 mmHg, and any deviation from a P_Baseline_ of 0 mmHg will over- or underestimate the true mean ICP.

BPEs occur whenever P_Baseline_ deviates from 0 mmHg. As all technical measurement methods carry some weaknesses, we considered differences in P_Baseline_ between Sensors 1 and 2 of 1–2 mmHg not to be BPEs. BPEs ≥5 mmHg were denoted as major since a falsely over- or underestimated mean ICP value of this magnitude may alter patient management. Each error large enough to falsely cross the accepted ICP threshold will either cause a potentially harmful clinical action, or lead to lack of action when actually necessary.

**Figure 2:**
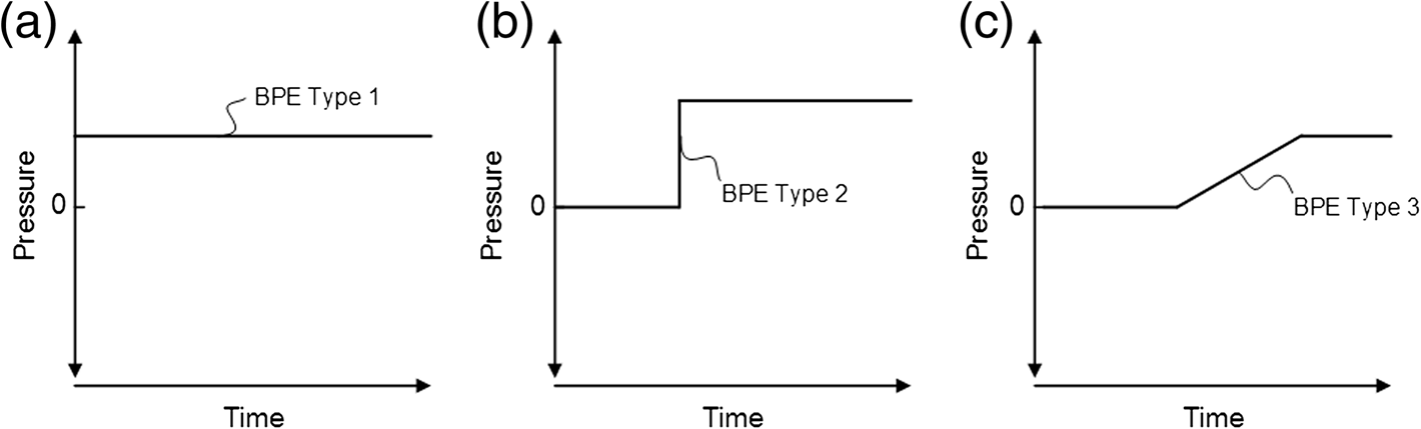
**Subtypes of baseline pressure errors (BPEs).** Illustration of the three types of BPEs: **(a)** Type 1: Constant BPE. **(b)** Type 2: BPE related to sudden shift in baseline pressure. **(c)** Type 3: BPE related to gradual drift of baseline pressure. While type 1 can be caused by wrong calibration from the manufacturer, or user-dependent erroneous zeroing, types 2 and 3 can be caused by electrostatic discharges, or various other causes.

We defined three separate types of BPEs (P_Baseline_ deviations), namely constant BPEs (Type 1), BPE related to sudden pressure shift (Type 2), and BPE related to gradual pressure drift (Type 3) (Figure 2). It was beyond the scope of this study to determine the relative contribution of the three sub-types of BPEs.

Determination of BPEs could be performed for 6-sec time windows that were accepted both for Sensors 1 and 2.

ICP waveforms with differences in MWA <0.5 mmHg were considered to be close to identical. Therefore, we analyzed only the 6-sec time windows with difference in MWA <0.5 mmHg. For each of these 6-sec time windows, differences between Sensors 1 and 2 in mean ICP ranging 2–5 mmHg, 5–10 mmHg, 10–15 mmHg, and 15–20 mmHg, were determined.

## Results

### Patients and ICP sensors

A total of 16 patients were enrolled in this study (Table 1). They were all hospitalized for aneurysmal SAH, and taken care of at the intensive care unit (ICU). No adverse effects of ICP monitoring were observed.

**Table 1:** Demographic data of the 16 patients included in the study, and distance between sensors 1 and 2

**Pat**	**Age**	**Gender**	**Distance between ICP sensors 1 and 2 (mm)**		
			**Axial**	**Coronal**	**Sagittal**
1	52	M	13	9	7
2	57	M	30	17	22
3	64	M	6	17	11
4	59	F	3	5	11
5	58	F	38	13	25
6	39	F	13	11	7
7	58	F	11	6	18
8	66	M	4	5	5
9	44	M	3	3	3
10	70	M	7	4	5
11	49	M	11	5	6
12	52	F	21	20	26
13	63	F	2	4	4
14	74	F	13	11	11
15	56	F	3	3	9
16	55	M	19	19	8
Median (Ranges)			11 (2–38)	8 (3–20)	9 (3–26)

**Figure 3:**
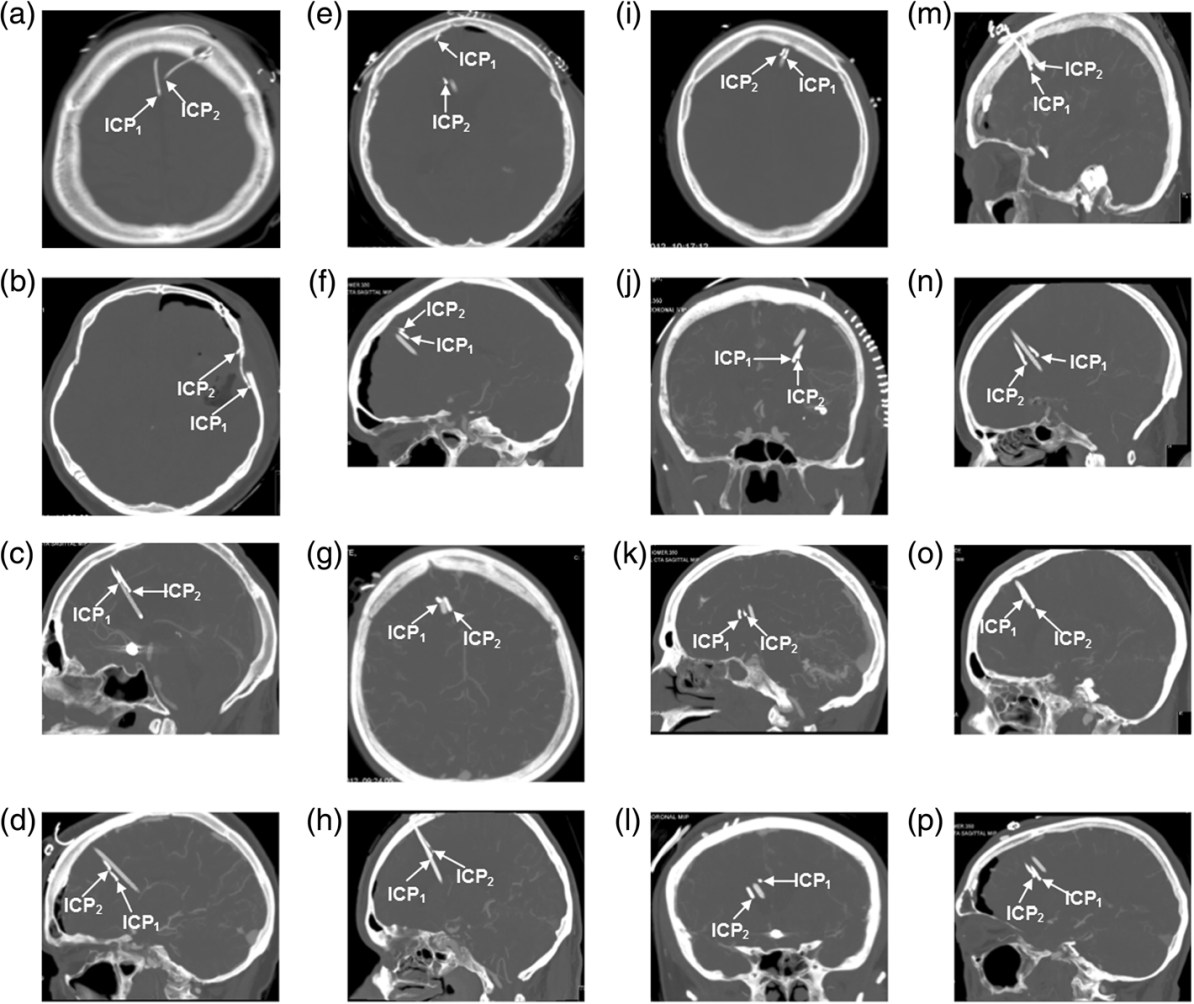
**Locations of ICP sensors 1 and 2 on cerebral computer tomography (CT).** The CT scans showing the two ICP sensors (ICP_1_/ICP_2_) in **(a)** Patient 1, **(b)** Patient 2, **(c)** Patient 3, **(d)** Patient 4, **(e)** Patient 5, **(f)** Patient 6, **(g)** Patient 7, **(h)** Patient 8, **(i)** Patient 9, **(j)** Patient 10, **(k)** Patient 11, **(l)** Patient 12, **(m)** Patient 13, **(n)** Patient 14, **(o)** Patient 15, and **(p)** Patient 16.

The distance between the ICP sensors are presented in Table 1, and measured axially a median of 11 (2–38) mm, coronally a median of 8 (3–20) mm and sagitally a median of 9 (3–26) mm. Figure 3 presents the cranial CT scans of the 16 patients showing the location of the ICP sensors. None of the ICP sensors seemed to be in direct contact with each other.

### Differences in mean ICP between Sensors 1 and 2

Major differences (>5 mm Hg) in mean ICP between Sensors 1 and 2 were observed in 7 of 16 patients (44%), including patients, 1, 2, 6, 8, 9, 13 and 16 (Table 2). In a significant number of patients, mean ICP of the two sensors deviated in different direction from a given threshold (Table 3). This was particularly evident in 11 of the 16 patients (69%; Patients 1, 2, 4, 6–10, 13, 15 and 16).

**Table 2:** Mean ICP recorded from sensor 1 and sensor 2

**Pat**	**Number of 6-sec TS observations**	**Mean ICP (mmHg; average ± std)**		**Difference mean ICP (mmHg; average ± std)**	**Percentage of 6-sec TS with difference in mean ICP:**		
		**Sensor 1**	**Sensor 2**	**Sensor 2 – Sensor 1**	**≥5 mmHg**	**≥10 mmHg**	**≥15 mmHg**
1	77,900	8.7 + 4.1	1.1 + 3.9	-7.6 ± 1.9	90	1	-
2	66,612	9.7 + 9.4	5.6 + 5.9	-4.1 + 9.6	65	14	10
3	133,387	5.4 + 4.8	6.0 + 4.8	0.6 + 1.2	-	-	-
4	84,355	7.2 + 4.1	8.2 + 4.0	0.9 + 0.6	-	-	-
5	41,708	7.4 + 5.8	8.1 + 5.4	0.8 + 0.7	-	-	-
6	47,972	4.4 + 3.4	9.5 + 3.2	5.1 + 0.4	55	-	-
7	127,305	6.9 + 4.3	7.4 + 3.9	0.4 + 1.6	1	-	-
8	147,216	9.5 + 4.9	4.8 + 4.0	-4.8 + 2.7	27	5	-
9	170,327	8.1 + 5.1	10.3 + 5.1	2.3 + 3.6	11	6	2
10	139,019	6.9 + 3.5	8.5 + 3.5	1.7 + 0.7	-	-	-
11	58,886	12.2 + 4.7	11.9 + 4.6	-0.4 + 0.9	-	-	-
12	123,334	11.2 + 3.8	11.0 + 3.7	-0.2 + 0.5	-	-	-
13	61,780	1.0 + 4.9	5.7 + 4.9	4.7 + 4.4	39	9	1
14	83,510	7.0 + 3.4	7.4 + 3.6	0.4 + 0.7	-	-	-
15	218,383	5.4 + 3.8	2.9 + 3.5	-2.5 + 1.0	2	-	-
16	112,501	12.1 + 6.0	1.4 + 5.9	-10.7 + 1.2	100	80	

**Table 3:** Distribution of observations wherein sensors 1 and 2 displayed values above/below certain thresholds

		**Percentage of combinations**		
**PatID**	**Number of 6-sec TS observations**	**Sensor 1 ≥10 mmHg/Sensor 2 <10 mmHg or Sensor 1 <10 mmHg/Sensor 2 ≥10 mmHg**	**Sensor 1 ≥15 mmHg/Sensor 2 <15 mmHg or Sensor 1 <15 mmHg/ Sensor 2 ≥15 mmHg**	**Sensor 1 ≥20 mmHg/ Sensor 2 <20 mmHg or Sensor 1 <20 mmHg/ Sensor 2 ≥20 mmHg**
**1**	77,900	31%	2%	-
**2**	66,612	37%	19%	12%
**3**	133,387	3%	1%	-
**4**	84,355	8%	1%	-
**5**	41,708	4%	2%	-
**6**	47,972	33%	3%	-
**7**	127,305	9%	2%	1%
**8**	147,216	35%	12%	2%
**9**	170,327	20%	7%	2%
**10**	139,019	14%	2%	-
**11**	58,886	3%	5%	2%
**12**	123,334	5%	3%	-
**13**	61,780	8%	2%	-
**14**	83,510	6%	1%	-
**15**	218,383	10%	1%	-
**16**	112,501	57%	15%	3%

### Differences in ICP wave parameters between Sensors 1 and 2

The percentage of accepted 6-sec time windows for these 16 patients was median 93% (ranges 40 – 100%; Table 4). There were only minor differences in the ICP wave parameters between Sensors 1 and 2 (Table 4). In particular, the differences in MWA between Sensors 1 and 2 were minor (Table 4).

**Table 4:** ICP wave parameters recorded from sensor 1 and sensor 2

**Pat**	**Number of 6-sec TS observations**	**Mean wave amplitude (MWA) (mmHg; average + std)**		**Mean wave rise time (MWRT) (sec; average ± std)**		**Mean wave rise time coefficient (MWRTC) (mmHg/sec; average ± std)**	
		**Sensor 1**	**Sensor 2**	**Sensor 1**	**Sensor 2**	**Sensor 1**	**Sensor 2**
1	50,641	3.4 ± 1.1	3.3 ± 1.1	0.22 ± 0.07	0.22 ± 0.07	19.3 ± 9.9	19.0 ± 9.9
2	26,373	3.3 + 1.2	3.9 + 0.9	0.23 + 0.08	0.22 + 0.09	17.0 + 7.5	22.8 + 10.8
3	132,424	4.6 + 1.6	4.9 + 1.5	0.13 + 0.02	0.13 + 0.02	39.7 + 11.9	42.1 + 11.8
4	78,521	6.8 + 1.4	6.8 + 1.4	0.27 + 0.04	0.27 + 0.04	27.5 + 9.4	27.5 + 9.3
5	40,333	9.9 + 2.7	9.9 + 2.7	0.20 + 0.03	0.20 + 0.03	52.1 + 14.8	52.2 + 15.0
6	29,816	3.6 + 0.7	3.5 + 0.7	0.20 + 0.07	0.20 + 0.07	22.1 + 8.4	22.1 + 8.5
7	121,786	6.1 + 1.9	6.3 + 2.0	0.20 + 0.04	0.20 + 0.04	32.4 + 9.7	33.0 + 9.5
8	140,666	5.2 + 1.4	5.2 + 1.4	0.18 + 0.05	0.18 + 0.05	33.3 + 9.3	32.9 + 9.0
9	151,794	5.3 + 2.1	4.9 + 2.3	0.16 + 0.06	0.17 + 0.08	40.9 + 13.1	37.4 + 17.0
10	136,594	6.2 + 1.1	6.1 + 1.1	0.18 + 0.04	0.18 + 0.04	38.2 + 9.6	37.8 + 9.7
11	57,819	6.6 + 2.1	6.5 + 2.1	0.18 + 0.04	0.18 + 0.04	40.2 + 15.1	40.0 + 15.0
12	122,988	6.2 + 2.0	6.2 + 2.0	0.21 + 0.03	0.21 + 0.03	30.3 + 8.3	30.3 + 8.3
13	44,746	3.7 + 0.8	3.7 + 0.9	0.23 + 0.06	0.23 + 0.06	19.6 + 10.6	20.4 + 10.8
14	75,821	5.8 + 1.0	5.9 + 1.0	0.21 + 0.05	0.21 + 0.05	31.3 + 10.4	31.5 + 10.5
15	188,848	3.3 + 1.2	3.3 + 1.2	0.24 + 0.05	0.24 + 0.05	15.1 + 5.8	14.9 + 5.8
16	104,993	5.6 + 1.9	5.6 + 1.9	0.16 + 0.06	0.16 + 0.06	42.8 + 16.8	42.6 + 17.0

### Occurrence of baseline pressure errors (BPEs)

Since BPE analysis required accepted 6-sec time windows from both ICP sensors, the median number of 6-sec time windows analyzed was 86,349 (range 5,987 – 185,697) (Table 5).

**Table 5:** Levels of BPE (difference in mean ICP between sensors 1 and 2 with close to identical waveform)

		**Levels of BPE (absolute values)**			
**Pat.**	**Number of 6-sec TS observations**^**a**^	**≥2 mmHg/<5 mmHg N (%)**	**≥5 mmHg/<10 mmHg N (%)**	**≥10 mmHg/<15 mmHg N (%)**	**≥15 mmHg/<20 mmHg N (%)**
**1**	49,664	1,115 (2%)	48,159 (97%)	389 (1%)	1
**2**	5,987	2,027 (34%)	3,576 (60%)	100 (2%)	256 (4%)
**3**	12,6481	3,351 (3%)	311	16	5
**4**	74,612	90			
**5**	39,938	551 (1%)			
**6**	29,613	12,819 (43%)	16,794 (57%)		
**7**	104,784	6,565 (6%)	100		
**8**	107,760	81,708 (76%)	17,120 (16%)	2,897 (3%)	325
**9**	122,850	2,406 (2%)	63	36	32
**10**	135,700	32,788 (24%)	30		
**11**	57,124	6,934 (12%)	126	1	
**12**	122,731	37			
**13**	42,802	28,677 (67%)	10,700 (25%)	3,305 (8%)	118
**14**	73,063	2,596 (4%)	3		
**15**	185,697	143,272 (77%)	4,415 (2%)	7	11
**16**	98,086	100	20,474 (21%)	77,512 (79%)	

BPE: Baseline Pressure Error. ^a^Including only 6-s TS with close to identical waveform, quantified as 6-sec TS with difference in MWA <0.5 mmHg. Numbers (and percentages) that we consider as major.

**Figure 4:**
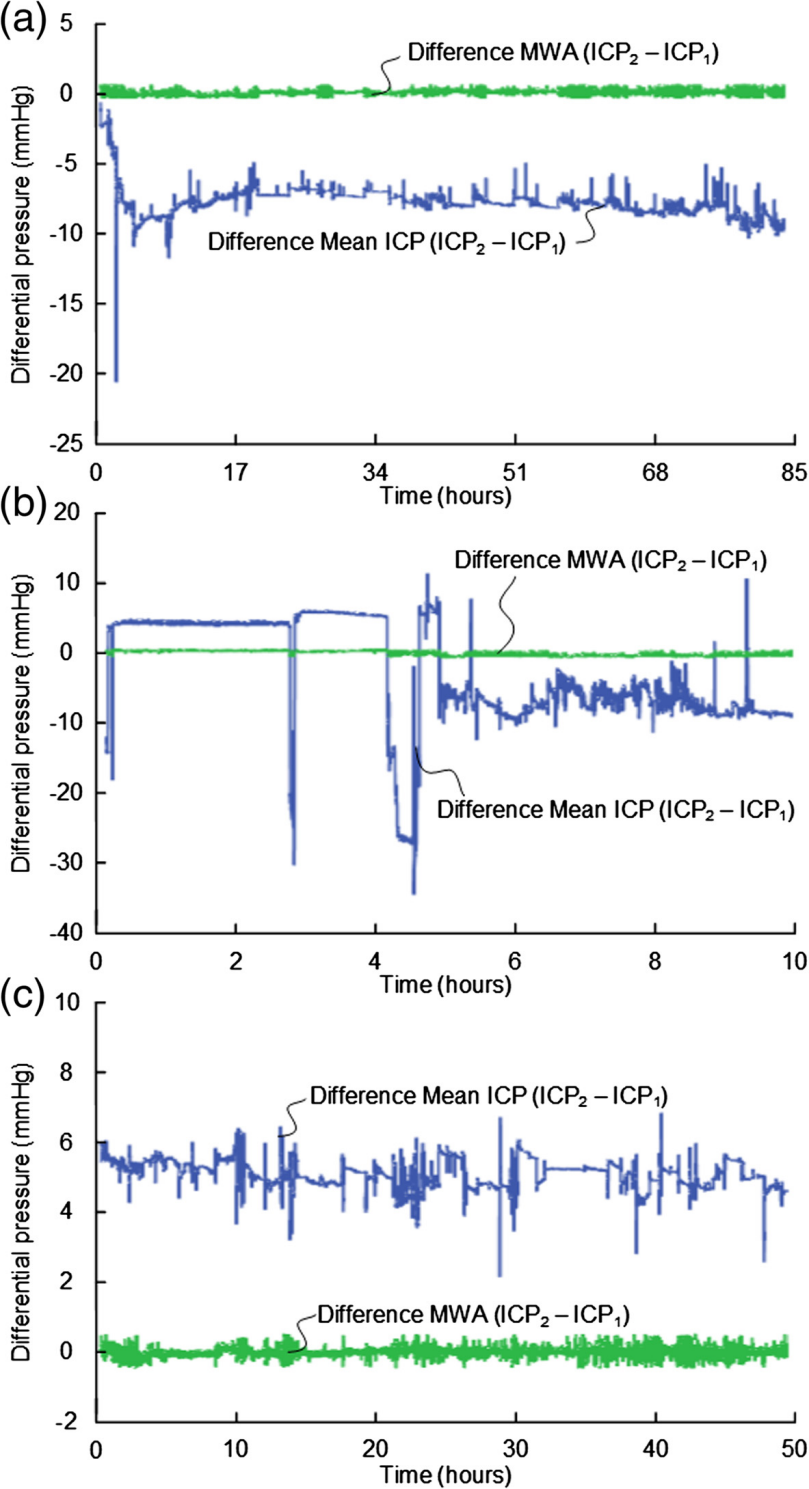
**Differential pressure trend plots of mean ICP/MWA of signals 1 and 2.** Differential pressure trend plots of **(a)** Patient 1, **(b)** Patient 2, and **(c)** Patient 6. The trend plots in blue reveal differences in mean ICP computed for consecutive 6-sec time windows (Mean ICP_Signal 2 –_ Mean ICP_Signal 1_), while the green plots show differences in MWA (MWA_Signal 2 –_ MWA_Signal 1_) of Signals 1 and 2, of the same 6-sec time windows. The presence of PBEs are indicated by major differences in mean ICP but with close to identical MWAs (differences in MWA <0.5 mmHg). **(a)** and **(b)** BPE type 2. **(c)** BPE type 1.

**Figure 5:**
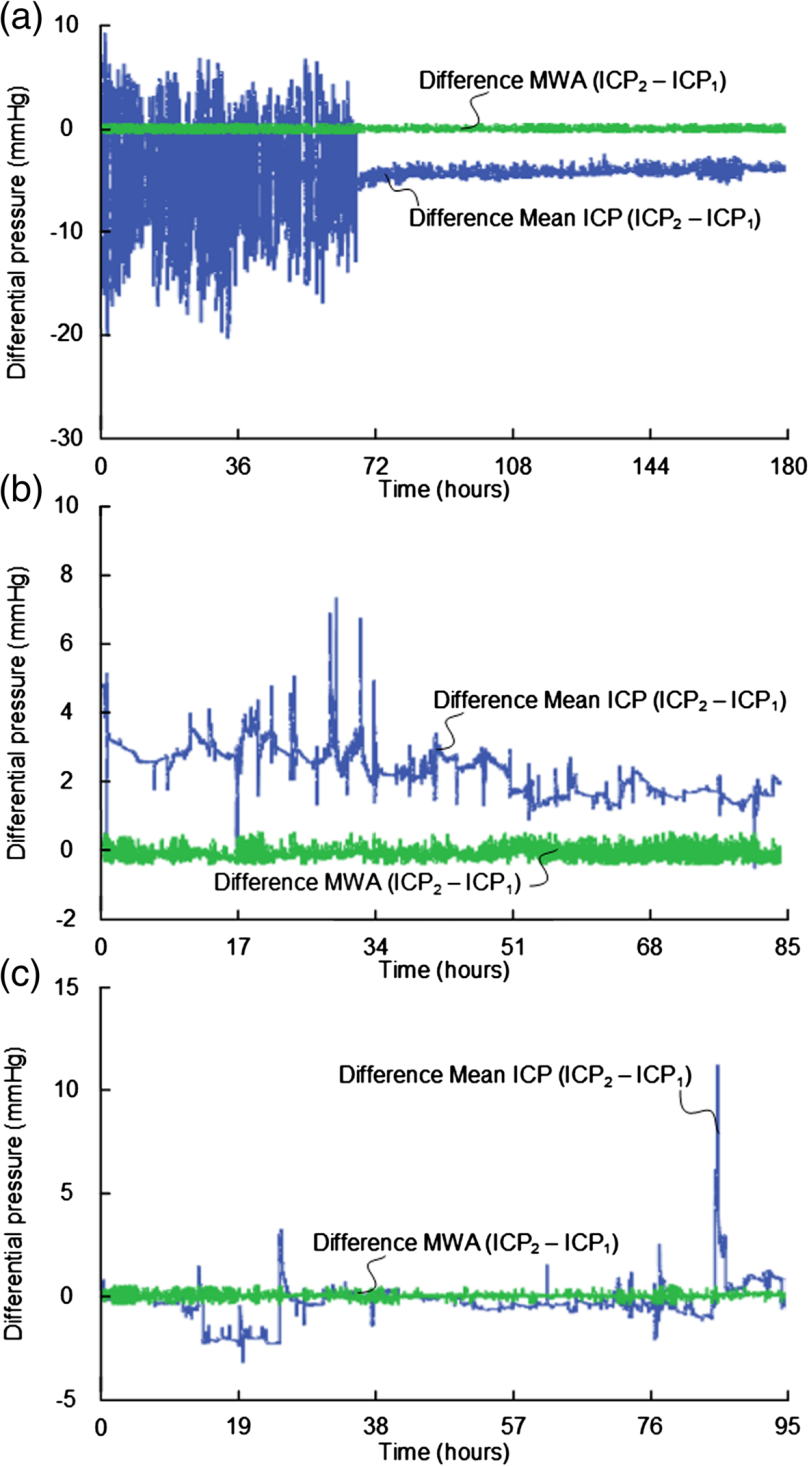
**Differential pressure trend plots of mean ICP/MWA of signals 1 and 2.** Differential pressure trend plots of **(a)** Patient 8, **(b)** Patient 10, and **(c)** Patient 11. The trend plots in blue reveal differences in mean ICP computed for consecutive 6-sec time windows (Mean ICP_Signal 2 –_ Mean ICP_Signal 1_), while the green plots show differences in MWA (MWA_Signal 2 –_ MWA_Signal 1_) of Signals 1 and 2, of the same 6-sec time windows. The presence of PBEs are indicated by major differences in mean ICP but with close to identical MWAs (differences in MWA <0.5 mmHg). **(a)** and **(c)** BPE type 2, **(b)** BPE type 1.

**Figure 6:**
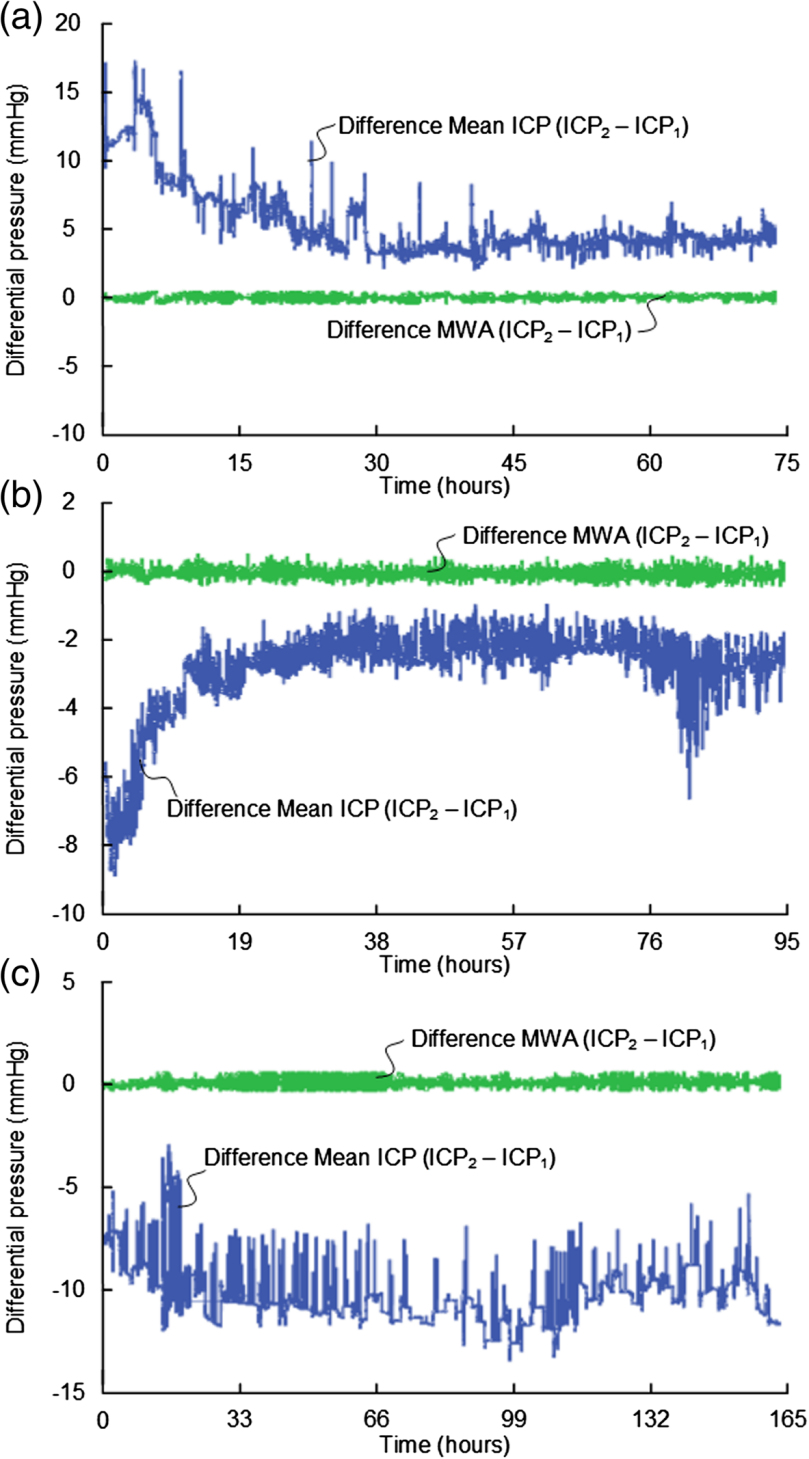
**Differential pressure trend plots of mean ICP/MWA of signals 1 and 2.** Differential pressure trend plots of **(a)** Patient 13, **(b)** Patient 15, and **(c)** Patient 16. The trend plots in blue reveal differences in mean ICP computed for consecutive 6-sec time windows (Mean ICP_Signal 2 –_ Mean ICP_Signal 1_), while the green plots show differences in MWA (MWA_Signal 2 –_ MWA_Signal 1_) of Signals 1 and 2, of the same 6-sec time windows. The presence of PBEs are indicated by major differences in mean ICP but with close to identical MWAs (differences in MWA <0.5 mmHg). **(a)** and **(b)** BPE type 3 and **(c)** BPE type 2.

We found significant BPEs in 9 of 16 patients (56%), including patients 1, 2, 6, 8, 10, 11, 13, 15 and 16 (Table 5). In these 9 patients, BPEs of 2–5 mmHg occurred in median 34% of observations (ranges 0-77%), while BPEs in the magnitude of 5–10 mmHg occurred in median 21% of observation (ranges 0-97%). Examples of BPEs in the 9 patients with severe BPEs are shown in Figures 4, 5, and 6.

In another 5 patients (31%; patients 3, 5, 7, 9 and 14), BPEs in the 2–5 mmHg range were seen in 1-6% of observations (median 3%; Table 5).

In the last 2 of 16 patients (13%; patients 4 and 12) BPEs of 2–5 mmHg constituted merely <1% of observations (Table 5).

## Discussion

The major finding of this study was that BPEs occur in a significant proportion of patients undergoing ICP monitoring. While BPEs lead to erroneous reading of the static pressure parameter mean ICP, the various ICP wave parameters are not affected by BPEs.

### The clinical significance of BPEs

Continuous monitoring of intracranial pressure (ICP) is a cornerstone in the surveillance of patients suffering traumatic brain injury or intracranial haemorrhages. Even though ICP monitoring has attracted the interest of neuroscientists for decades, the role of BPEs has not been discussed even in recent reviews on ICP monitoring [8-10].We see two possible reasons for why the issue of BPEs previously has received little attention. Firstly, single ICP wave analysis is not conventionally performed during ICP monitoring. Secondly, measurements from two simultaneous ICP sensors are rarely done. This makes it very difficult to identify BPEs as the cause of alterations in the mean ICP.

### Monitoring from two separate ICP sensors simultaneously

To our knowledge, Fernandes et al. [11] were the first to report major differences in mean ICP when monitoring simultaneously from two separate sensors (one Codman and one Camino sensor). While they demonstrated sudden shifts in mean ICP, they did not monitor the ICP waveform. In 2006, Eide [5] confirmed their observations, reporting marked differences in mean ICP when recording simultaneously from one Codman and one Camino sensor. These differences in mean ICP were explained by BPEs as the differences in mean ICP were associated with close to identity in the ICP wave parameters [5]. In Eide’s study, the comparison was based on automatic identification of heartbeat induced single ICP waves. In a subsequent study [12], Eide et al. demonstrated how the relationship between mean ICP and the MWA suddenly changed due to abrupt shifts or drifts in this relationship. In that study, however, recordings were carried out using only one ICP sensor and consequently only indirect evidence of BPEs were obtained. The same phenomenon was also observed when Eide [13] and Eide and Sorteberg [14] monitored simultaneously in two separate ICP sensors, one placed in the brain parenchyma and the other in the epidural space compartment. In these two studies, however, the differences in mean ICP could possibly have been caused by monitoring from two separate intracranial compartments.

In a recent report [6], we reviewed our experience with monitoring simultaneously from two separate ICP sensors, demonstrating that BPEs of drift and shift types occur clinically. We showed that BPEs may occur in solid sensors (Codman), air-pouch sensors (Spiegelberg) as well as fluid sensors (Edward’s Life Science). In fact, all the various ICP sensors we have tested, the Raumedic Neurovent P [15,16], the Codman [17], the Camino [18] and the Spiegelberg [19] sensors, have been sensitive to BPEs. When monitoring ICP through an EVD, BPEs may in addition be created by imperfect fluid connection caused by air bubbles and debris, or through movement of the sensor position (height) relative to the measurement site.

### Monitoring of static – versus dynamic intracranial pressure parameters

The principles for measuring static mean ICP and dynamic ICP parameters such as the mean wave amplitude (MWA) and the mean wave rise time (MWRT) are fundamentally different. While the static mean ICP is calculated by always relating to a defined and constant value (the baseline reference pressure/zero pressure level) (Formula 1), the dynamic intracranial pressure parameters are obtained within the pressure signal itself, referring to pressure values within one single heartbeat. The size of the ICP wave during a heartbeat will hence be 4 mmHg independent of whether the ICP during that heartbeat fluctuates between e.g. 8 and 4 mmHg or between 22 and 18 mmHg. In contrast, if the baseline ICP reference pressure changes, the mean ICP and every pressure index wherein the mean ICP is incorporated, e.g. indices of pressure volume-reserve capacity and pressure-reactivity [20], becomes erroneous from that moment on. On the other hand, the dynamic ICP parameters, except those calculated from the one - or the very few heart beats during which a BPE occurs, remain unchanged.

### The nature and causes of BPEs

By definition, BPEs are baseline pressures deviating from zero pressure, whether measured in mmHg or Pascal (Pa). However, there are no uniformly accepted ways to determine BPEs, and consequently this topic has not been addressed in the literature. In this study, we defined BPEs as significant differences in mean ICP between two sensors despite close to identical ICP waveforms. This definition concurs with our previous work [5,6]. Evidence for the close to identical ICP waveform is based on visual assessments of raw signals with known MWA values. As the upper normal thresholds level for MWA is 4–5 mmHg, there are solid arguments for using 6-sec time windows with difference in MWA <0.5 mmHg as indicative of identical ICP waveform.

In an in-vitro study, Eide and Bakken in 2011 [21], showed that the Raumedic and Codman ICP sensors, are sensitive to electrostatic discharges (ESDs). In this study, BPEs in the forms of sudden pressure shifts and pressure drifts were seen following ESDs. Furthermore, the BPEs were of such magnitudes that given a similar change in mean ICP in a clinical setting, it could alter patient management. Other authors recently confirmed the sensitivity of the Raumedic and Codman ICP sensors to ESDs [22].

Causes for BPE may be different when recording from a fiberoptic ICP sensor, a solid sensor based on the whetstone bridge principle, or from an air-pouch type of ICP sensor. There are several potential sources of BPEs. Several technical components are required for ICP monitoring (sensor, cable, transducer, display), all of which may be origos of BPEs. The transducers provided by different manufacturers may have different susceptibility to cause BPEs, though this is an open question.

This study demonstrates that BPEs may extensively influence the static mean ICP readings (Table 5). Furthermore, the BPEs were at times of such magnitudes that the erroneous mean ICP readings may affect patient management if not recognized. This finding may have a profound bearing on the value of ICP monitoring in critically ill patients.

The dynamic ICP wave parameters are independent of a baseline pressure, and are therefore not affected by BPEs [5]. Consequently, we see monitoring of dynamic ICP parameters at present as the best way of identifying possible BPEs and thereby limiting their impact. A requirement for proper ICP wave monitoring is, however, correct single wave identification to assure quality control.

The future of regular ICP monitoring with one sensor even in the presence of single wave analysis, remains to be determined. We do not yet have an established methodology for determining BPEs from one signal only. A possible strategy could be to determine how mean ICP and MWA relates during ongoing monitoring [12]. Hence, a sudden change in mean ICP not accompanied by a change in the ICP wave amplitude should alert the clinician to a technical rather than a biological problem. Another option could be to manage patients solely based on dynamic ICP waves findings, disregarding the static mean ICP [1].

### The concept of *State of the Art*

For a medical device, the concept of *State of the Art* implies that the best available technology is incorporated. A medical device for monitoring of ICP shall hence provide as correct information as possible about the ICP, using the best available technology. Today’s *State of the Art* in ICP monitoring relies on the determination of ICP relative to a baseline pressure. However, the high frequency of BPEs seen in a large proportion of patients in the present study (Table 5) shows that current *State of the Art* requires a change. Given that ICP wave monitoring avoids the BPEs, ICP wave monitoring should become the new *State of the Art*.

## Conclusions

Baseline Pressure Errors (BPEs) occur in a significant proportion of patients undergoing ICP monitoring. They lead to erroneous scoring of the various static ICP parameters whereas the dynamic ICP parameters remain correct and stable. The magnitude of the BPEs is such that they may influence treatment, and hence potentially diminish the value of mean ICP monitoring. The results demonstrate that measurements of ICP against a baseline pressure do not comply with the concept of State of the Art. Monitoring of the ICP waves ought to become the new *State of the Art* as they are not influenced by BPEs.

## Abbreviations

BPE: Baseline pressure error; CT: Computer tomography scanning; dP: Amplitude; dT: Rise time; dP/dT: Rise time coefficient; EVD: External ventricular drainage; ICP: Intracranial pressure; MWA: Mean ICP wave amplitude; MWRT: Mean wave rise time; MWRTC: Mean wave rise time coefficient; SAH: Subarachnoid haemorrhage; SW: Single wave.

## Competing interests

AS, TRM and WSO report no conflicts of interest. PKE has financial interest in the software company (dPCom A/S) that manufactures the software (Sensometrics® Software), which was used for digital recording of the continuous pressure signals in this study.

## Authors’ contributions

All authors contributed to conception and design, acquisition and interpretation of data. PKE contributed the bulk of the drafting of the manuscript and AS, TRM and WSO contributed with thorough editing of the manuscript. All authors have read and approved the final manuscript.
